# Supplementary material for: A Human Stem Cell-Derived Neurosensory–Epithelial Circuitry on a Chip to Model Herpes Simplex Virus Reactivation
Source: Biomedicines. 2022 Aug 24;10(9):2068. doi: 10.3390/biomedicines10092068 (PMC9495731; doi:10.3390/biomedicines10092068)
Supplement: Supplementary file 1 [file biomedicines-10-02068-s001.zip › biomedicines-1883814-supplementary.pdf]

SUPPLEMENTARY MATERIALS

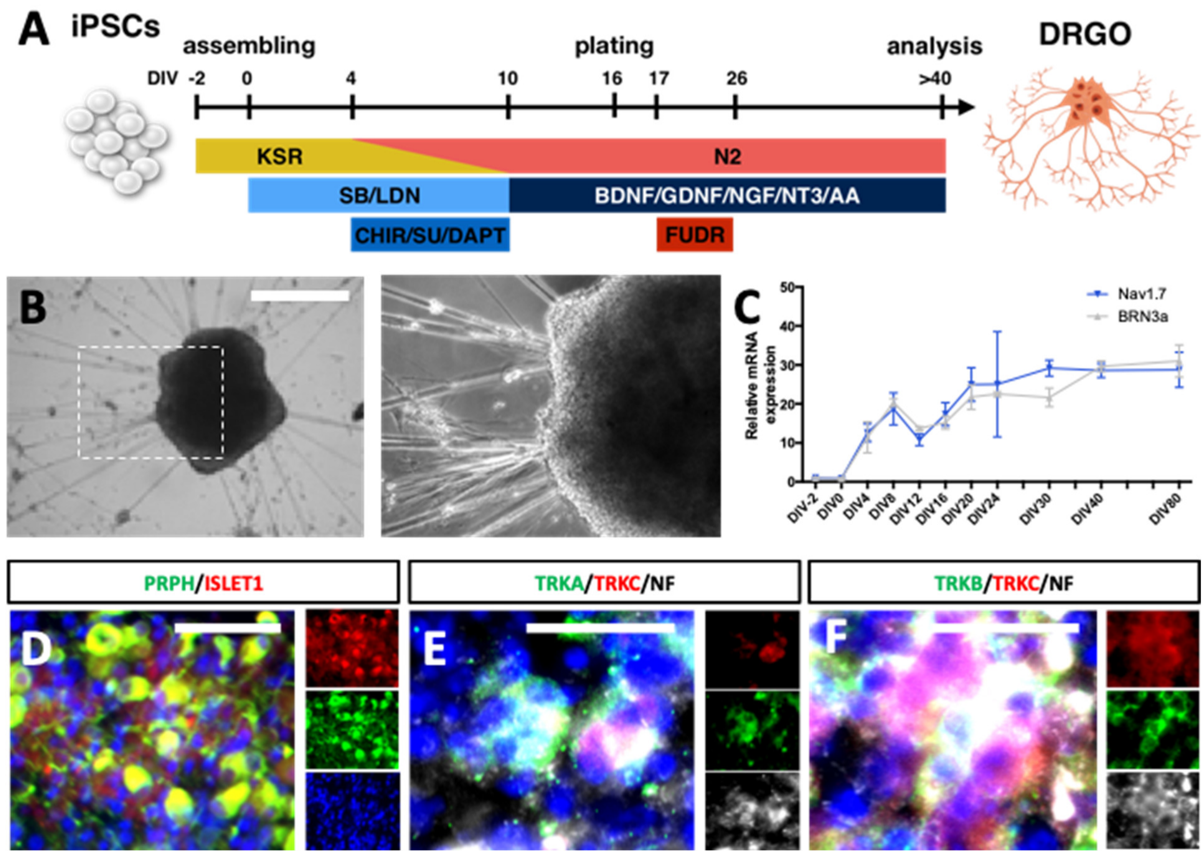

Figure S1. iPSC-derived organoid resembling primary dorsal root ganglia (DRGOs). (A) Illustration of the 3D culture system and sequential exposure to small molecules over time to obtain DRGOs. (B) Images of DRGO at DIV 40 with star-like web of neurites projections around the central mass and magnification. Scale bar, 500μm. (C) Quantitative analysis of BRN3A and NAV1.7 transcript levels during DRGO differentiation. Expression levels are normalized to actin. Mean ± SD, *n* = 3 independent experiments, 8-12 organoids/line/experiment. (D) Immunocytochemistry in DRGOs at DIV 40 for proteins localized along neuronal projections

peripherin, PRPH and the sensory neuron-specific transcription factors ISL1. Scale bar, 50µm. (E) Immunocytochemistry on DIV 40 DRGOs of Neurotrophic receptor tyrosine kinases (NTRK) 1/2/3 combination distinguishing the nociceptive (TRKA+/TRKC-, red arrowheads). Scale bar, 10µm. (F) Immunocytochemistry to discriminate mechanoreceptive (TRKB+/TRKC+, yellow arrowheads) and proprioceptive (TRKB-/TRKC+, red arrowheads) neuronal subtypes. Scale bar, 10 µm.

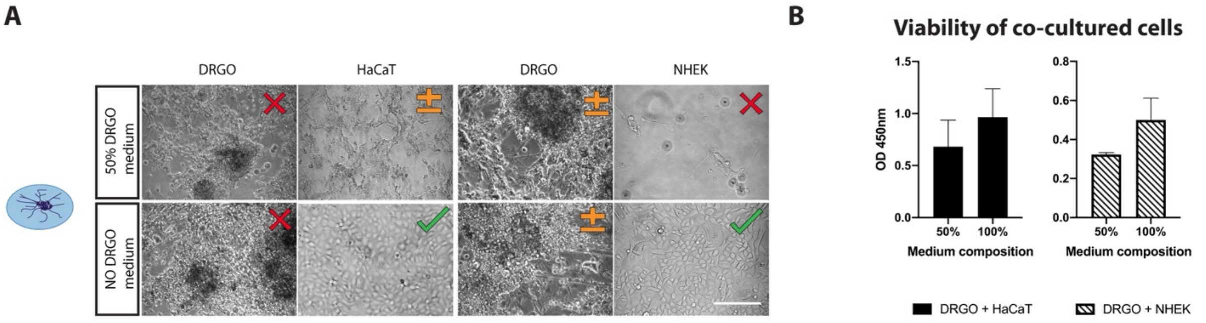

Figure S2. **Unpattern co-culture of organoids and keratinocytes.** (A) Bright-field microscopy images (20x magnification) of direct co-cultures of organoids (DRGOs) and both immortalized (HaCaT) and primary (NHEK) keratinocytes. Scale bar, 100 µm. (B) Cell proliferation assay to test DRGOs, HaCaT and NHEK viability. Mean + SD. "X" indicates that culture conditions are detrimental for cell viability and morphology, "±" indicates that viability of cells is around 50%, "∨" indicates that media allow to retain good cell morphology and viability

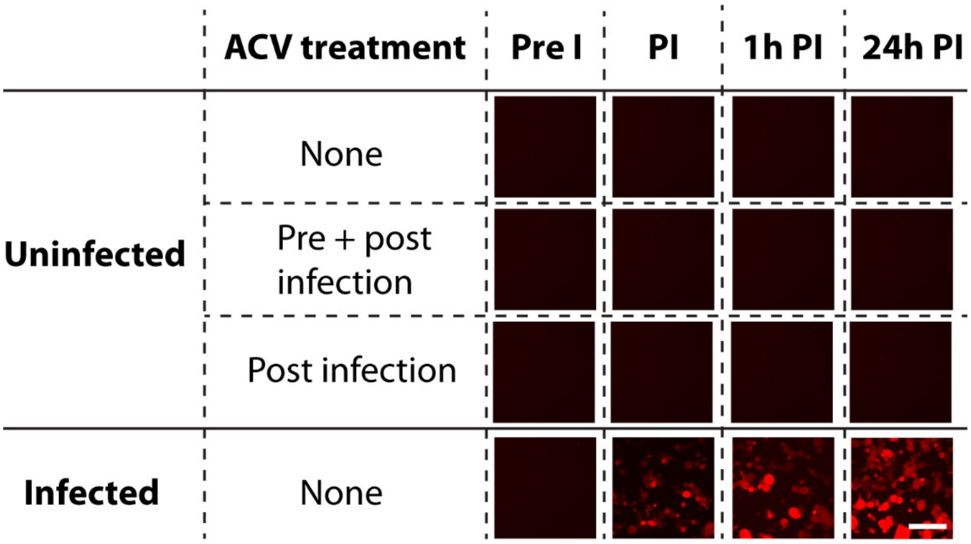

Figure S3. Microfluidics chip validation. Chip supernatants collected at different time points tested on sensor cells for the presence of infective virus particles due to passive diffusion (lytic infection = red fluorescent signal). (Pre I = before infection; PI = post-infection; 1h PI = 1 h post-infection; 24h PI = 24 h post-infection). Scale bar, 500 µm.

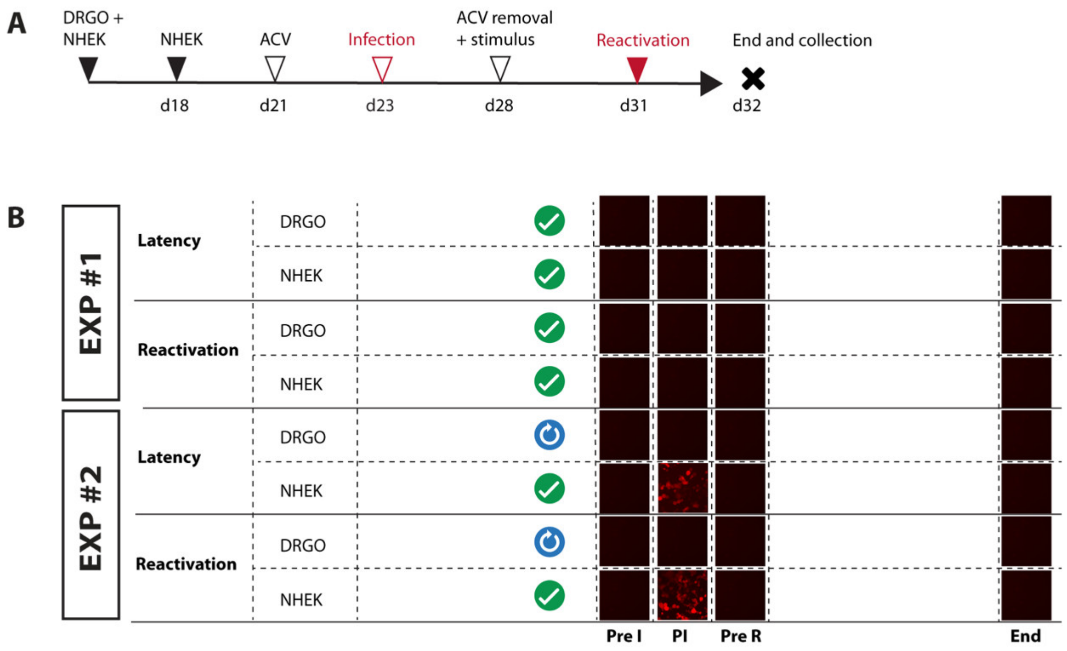

Figure S4. Reactivation protocol in microfluidics chip. (A) Schematic representation of latency establishment and reactivation protocol used in chip EXP #1 and #2. (B) Supernatants from different chambers and time points were tested on sensor cells as well (lytic infection = red fluorescent signal). Green tick indicates ACV addition to culture medium immediately after virus adsorption, blue arrow is for ACV delayed addition. (Pre I = 1 h before infection; PI = 1 h post-infection; Pre R = before reactivation, 5 DPI; End = end of the experiment, 8 DPI). Scale bar, 500 µm, n = 6 independent experiments for each tested condition.

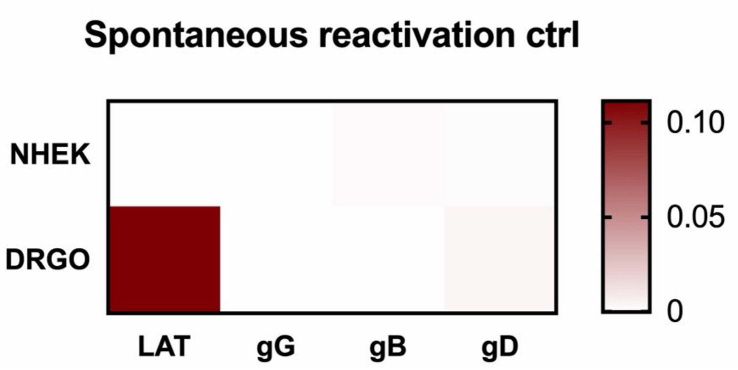

Figure S5. Control of spontaneous reactivation. Heatmap showing virus gene expression analysis of NHEK and DRGOs during latency without ACV addition to culture medium (dark red= high expression; white = no expression).
